# Supplementary material for: Preparation of Functional Nanoparticles-Loaded Magnetic Carbon Nanohorn Nanocomposites towards Composite Treatment
Source: Nanomaterials (Basel). 2023 Feb 23;13(5):839. doi: 10.3390/nano13050839 (PMC10005593; doi:10.3390/nano13050839)
Supplement: Supplementary file 1 [file nanomaterials-13-00839-s001.zip › nanomaterials-2212410-supplementary.pdf]

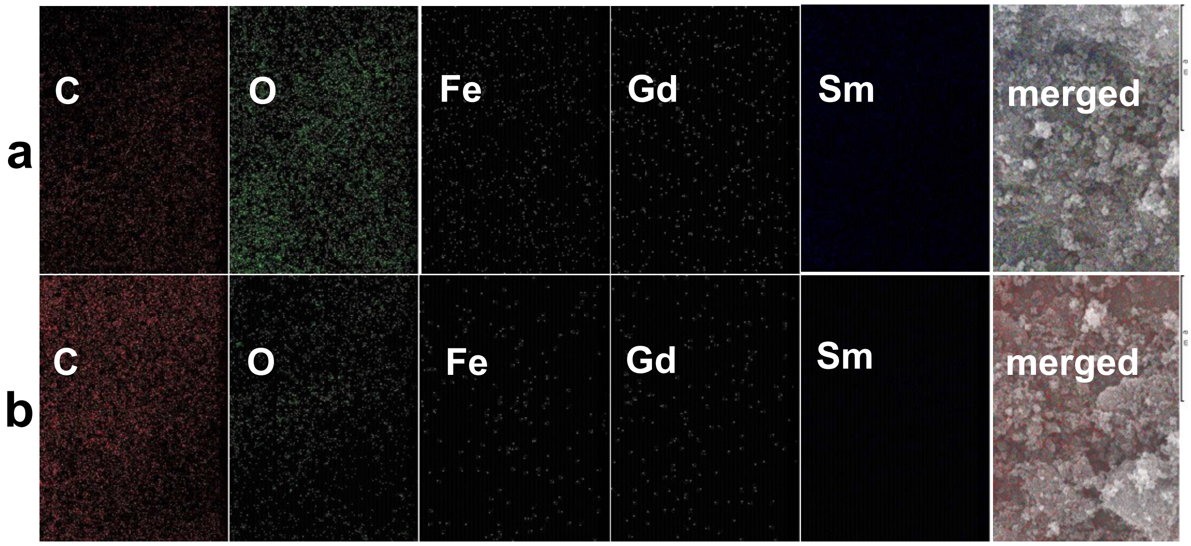

Figure S1. Elemental mapping of (a) CNH/Fe_3O_4/Sm_2O_3/Gd_2O_3 and (b) CNH/Fe_3O_4@Cdot/Sm_2O_3/Gd_2O_3.
